# Quantifying the efficacy of an automated facial coding software using videos of parents

**DOI:** 10.3389/fpsyg.2023.1223806

**Published:** 2023-07-31

**Authors:** R. Burgess, I. Culpin, I. Costantini, H. Bould, I. Nabney, R. M. Pearson

**Affiliations:** ^1^The Digital Health Engineering Group, Merchant Venturers Building, University of Bristol, Bristol, United Kingdom; ^2^The Centre for Academic Mental Health, Bristol Medical School, Bristol, United Kingdom; ^3^Florence Nightingale Faculty of Nursing, Midwifery and Palliative Care, King’s College London, London, United Kingdom; ^4^The Medical Research Council Integrative Epidemiology Unit, University of Bristol, Bristol, United Kingdom; ^5^The Gloucestershire Health and Care NHS Foundation Trust, Gloucester, United Kingdom; ^6^The Department of Psychology, Manchester Metropolitan University, Manchester, United Kingdom

**Keywords:** automated facial coding, FaceReader, facial expressions, parenting, ALSPAC

## Abstract

**Introduction:**

This work explores the use of an automated facial coding software - FaceReader - as an alternative and/or complementary method to manual coding.

**Methods:**

We used videos of parents (fathers, *n* = 36; mothers, *n* = 29) taken from the Avon Longitudinal Study of Parents and Children. The videos—obtained during real-life parent-infant interactions in the home—were coded both manually (using an existing coding scheme) and by FaceReader. We established a correspondence between the manual and automated coding categories - namely Positive, Neutral, Negative, and Surprise - before contingency tables were employed to examine the software’s detection rate and quantify the agreement between manual and automated coding. By employing binary logistic regression, we examined the predictive potential of FaceReader outputs in determining manually classified facial expressions. An interaction term was used to investigate the impact of gender on our models, seeking to estimate its influence on the predictive accuracy.

**Results:**

We found that the automated facial detection rate was low (25.2% for fathers, 24.6% for mothers) compared to manual coding, and discuss some potential explanations for this (e.g., poor lighting and facial occlusion). Our logistic regression analyses found that Surprise and Positive expressions had strong predictive capabilities, whilst Negative expressions performed poorly. Mothers’ faces were more important for predicting Positive and Neutral expressions, whilst fathers’ faces were more important in predicting Negative and Surprise expressions.

**Discussion:**

We discuss the implications of our findings in the context of future automated facial coding studies, and we emphasise the need to consider gender-specific influences in automated facial coding research.

## 1. Introduction

Manual coding is a method of analysing operationally-defined behaviours from observational data, allowing researchers to identify subtle behaviours and analyse changes over time. As observations are often coded at a high temporal resolution, manual coding is a rigorous and yet time consuming process. Coding may also be subject to inherent human biases, for example, via coder fatigue or previous coding experience (e.g., biassed by recent behaviours observed). Thus, it is advantageous to explore faster and potentially less biassed alternatives. For analysing facial expressions, an alternative approach is offered by automated facial coding (AFC) via software or other computational techniques. This method provides rapid, detailed, objective classification of expressions (which may help to reduce human biases; Skiendziel et al., 2019).

In this study, we specifically focus on evaluating the performance of Noldus FaceReader (Noldus, 2014), an automated facial coding software, in the context of parent-interaction videos captured within home settings. Our evaluation involves quantifying the facial detection rate of the software, and exploring the relationship between automated and human coding, with an emphasis on gender differences. It should be noted that throughout this work, we assume that the identified participant gender is equivalent to the participant’s biological sex at birth. By specifically examining gender, we seek to understand potential variations in the software’s performance and the agreement between automated and human coders based on gender-specific expressions. A preliminary version of this study has been previously reported (Burgess et al., 2022). By conducting this comprehensive evaluation, we aim to enhance our understanding of the capabilities and limitations of automated facial coding in the analysis of parent facial expressions, providing valuable insights for researchers and practitioners in the field.

### 1.1. Automated facial coding for emotion recognition

FaceReader is a general-purpose automated facial coding (AFC) software developed by Noldus, which employs a three-step process—face finding, modelling, and classifying—to accurately determine facial expressions (Den Uyl and Van Kuilenburg, 2005; Noldus, 2014). It offers classification into eight distinct expressions, including Happy, Sad, Angry, Scared, Surprised, Disgusted, Neutral, and Contempt. The software has been validated and found to outperform human coders, correctly identifying 88% of expressions compared to 85% by humans (Lewinski et al., 2014). FaceReader demonstrates high accuracy in classifying various expressions, with rates reported at 94% for Neutral, 82% for Scared, and other studies reporting performance rates ranging from 80 to 89% (Den Uyl and Van Kuilenburg, 2005; Terzis et al., 2013; Skiendziel et al., 2019).

Given its robust performance, FaceReader is a suitable choice for evaluating facial expressions in our study. Its reputation and widespread adoption in the research community make it a benchmark for comparison against other AFC methods. By evaluating FaceReader’s performance, we contribute to the literature on the validity and limitations of AFC. Moreover, previous works have already examined the comparison between manual and automated facial coding using FaceReader, revealing varying levels of agreement for different expressions (Terzis et al., 2010, 2013).

In many studies, authors analysed videos made in regulated contexts, e.g., with controlled lighting and backgrounds, and no other people present (Benţa et al., 2009; Danner et al., 2014; Brodny et al., 2016). However, these structured recordings are not representative of real-life conditions, and it is important to assess whether AFC algorithms can learn to detect expressions in more uncontrolled environments. In this work, we looked to evaluate how well this can be achieved by FaceReader, using videos within the home.

Recent advances in AFC have expanded our ability to capture and analyse facial expressions, enabling a deeper understanding of true emotional expressivity. Recent literature has addressed the limitations of earlier studies that primarily relied on standardised and prototypical facial expressions for validation. Büdenbender et al. (2023), Höfling et al. (2022), and Sato et al. (2019) explored the application of AFC in untrained participants who performed posed expressions, highlighting the challenges and potential in coding their facial expressions, and emphasising the importance of incorporating more naturalistic expressions in training AFC models. Furthermore, Höfling et al. (2021) investigated the differentiation of facial expressions in various social interaction scenarios, demonstrating the efficacy of FaceReader in the mimicking condition and the superiority of electromyogram (EMG) measures in passive viewing and inhibition conditions. Notably, Höfling et al. (2020) compared the sensitivity of FaceReader to established psychophysiological measures and found comparable results for pleasant emotions, but limitations in distinguishing between neutral and unpleasant stimuli. Similarly, Küntzler et al. (2021) evaluated multiple facial emotion recognition systems, including FaceReader, and showed accurate classification for standardised images but decreased performance for non-standardised stimuli. These findings underscore the need for improved AFC models that can handle more complex and realistic expressions. Moreover, Höfling and Alpers (2023) demonstrated that AFC can predict self-reported emotion. In the context of naturalistic observations, Gómez Jáuregui and Martin (2013) applied FaceReader to analyse a dataset of acted facial expressions under uncontrolled conditions and found that the software could not accurately classify any expression. Contrasting with this result, Krishna et al. (2013) achieved an expression classification accuracy of 20.51% using different automated methods with the same dataset, raising further questions about the performance of FaceReader in real-life recordings. Collectively, these studies contribute to the growing body of literature on the validity, limitations, and applications of AFC in understanding emotional facial expressions in diverse contexts, emphasising the importance of advancing AFC models to effectively capture and interpret facial expressions in realistic settings.

Several previous studies using FaceReader have used videos that were recorded using laptop webcams (Danner et al., 2014; Booijink, 2017; Talen and den Uyl, 2022), as videos captured in this way provide a direct view of the participant’s face. However, webcams (or any stationary cameras) are not the optimum choice for capturing facial expressions during naturalistic observations. This is because real-life interactions are more dynamic, often involving multiple people, body positions, and complex movements. Wearable headcams offer an alternative approach for capturing facial expressions in this kind of setting, as they offer a first-person perspective. Wearable headcams have been shown to reliably capture ecologically valid behaviours during parent-infant interactions (Lee et al., 2017). We have not identified any studies using FaceReader to analyse videos taken using wearable headcams during natural interactions.

### 1.2. Automated facial coding, parenting, and gender

Several studies have revealed valuable insights into the capabilities and applications of automated facial coding techniques in the field of parenting. Messinger et al. (2009) found high associations with manual coding and positive emotion ratings, whilst Mahoor et al. (2009) measured natural facial expressions with good agreement to human coding. Haines et al. (2019) used automated coding to analyse caregiver expressions, revealing insights on emotion dysregulation. These studies highlight the effectiveness of automated facial coding in understanding parent–child interactions and emotions.

So far, very few studies have evaluated the use of FaceReader in a parent-infant context. Karreman and Riem (2020) used webcams to record mothers’ reactions to images of unknown infants, and used the software to analyse their facial expressions. Lyakso et al. (2021) used FaceReader to quantify mother and infant facial expressions across various scenarios (e.g., mother-infant interactions, infant–infant interactions); all recordings in this work were made using a handheld video camera, and almost all interactions took place in naturalistic environments with uncontrolled lighting. The authors also excluded videos if the participants were not facing the camera (Lyakso et al., 2021). Aside from these studies (Karreman and Riem, 2020; Lyakso et al., 2021), we found no other literature evaluating maternal expressions using FaceReader, and we found no work either using FaceReader to evaluate paternal expressions, or drawing comparisons based on parent gender.

It is not surprising that there have not yet been any studies using FaceReader to evaluate paternal facial expressions, as father-infant interactions are rarely studied. A systematic review (*n =* 26) on parental facial expressions in family interactions found that less than half of the studies included fathers as participants (Hudon-ven der Buhs and Gosselin, 2018). As such, whilst research into parent behaviours in the mother-infant dyad is plentiful, less is known about the fathers and infants (Kokkinaki and Vasdekis, 2015). In a wider context, gender differences in emotional expressiveness have been widely accepted (Hall, 1990; Brody and Hall, 2008). Men have been shown to be less emotionally expressive than women (Campos et al., 2013), and to display less intense happy expressions (Oveis et al., 2009). A widely cited meta-analysis (*n =* 162 studies) of sex differences in smiling frequencies found that women smile more than men (LaFrance et al., 2003). By studying facial expressions—via comparisons of mother-infant and father-infant interactions—we can improve our understanding of gender specific differences in emotional communication. Whilst there has been little to no research into using automated facial coding to explore the differences between maternal and paternal expressions, some work has been carried out to evaluate gender differences more broadly. Software validation work concluded that the FaceReader software identified female facial expressions better than male (Lewinski et al., 2014). Another study found that FaceReader recognised Surprised and Scared better in males, and Disgusted and Sad better in females (Terzis et al., 2010).

Additional work is required to evaluate gender differences in parent expressions using AFC methods; exploring the existing studies linking automated facial coding to parenting and gender is important because it allows us to gain insights into how the method can be used to understand emotional expressions in the context of parental interactions and gender-specific differences, contributing to a more comprehensive understanding of emotional communication and dynamics in these areas.

### 1.3. Research aims

Overall, advances in AFC have greatly improved our understanding of facial expressions, overcoming limitations of previous studies that focused on standardised expressions. Studies involving untrained participants have revealed the challenges and potential of coding more naturalistic expressions. AFC has effectively differentiated facial expressions in diverse social scenarios and provided insights into self-reported emotions. However, there remains a critical gap in applying AFC to real-life conditions and limited research on parental facial expressions, specifically regarding differences between mothers and fathers.

To address these gaps, the present study evaluated FaceReader’s performance using videos of naturalistic parent-infant interactions. We used contingency analyses to investigate the detection rate of the software and the concordance between automated and manual facial coding, and employed logistic regression to further explore the relationship. The influence of parent gender on expression predictions was also explored. As such, our study is structured around three primary aims:

(A1):Assess the facial detection rate of the software.
(A2):Quantify the agreement between automated and manual facial coding: by assessing the concordance between the two methods, and using the automated outputs to predict the manual coding.
(A3):Investigate the influence of parent gender on the relationship between manual and automated facial coding, by analysing combined datasets as well as separate datasets for fathers and mothers.

To address these aims, we sought to examine parent facial expressions during interactions with their infants in real-life settings. We coded videos of parent-infant interactions using both manual coding via the Observer software (Noldus, 1991), and AFC via the FaceReader software (Noldus, 2014). The videos were manually coded for a previous project, according to the mutually exclusive expressions: Smile, Positive, Neutral/Alert, Negative, Surprise, Mock Surprise, Disgust, Woe face, None of the Above and Face not Visible. Conversely, FaceReader classifies expressions according to the following: Happy, Sad, Neutral, Angry, Surprised, Disgusted, Scared and Contempt. As the two sets of expressions are not an exact match, we created four mappings between the manual and automated expressions—Positive, Neutral, Negative and Surprise—as shown in Table 1. These mappings were used to address the concordance question in (A2), and as target variables for the predictive models used to address (A2) and (A3). The mappings indicate the expected relationships between the manual and automated codes; for example, we expect that Surprise is closely linked to FaceReader estimations of Surprise, and we expect that the manual codes Smile and Positive are linked to FaceReader estimations of Happy. Note that the manual codes None of the Above and Woe Face did not contribute to any mapping in Table 1, so analyses for these expressions were not included in this work.

**Table 1:** Approximate mapping between manual and FaceReader expressions.

Manual expression(s)		FaceReader expression(s)	Mapping
Neutral/Alert	→	Neutral	Neutral
Smile + Positive	→	Happy	Positive
Negative + Disgust	→	Sad + Angry + Disgusted + Scared + Contempt	Negative
Surprise + Mock surprise	→	Surprised	Surprise
None of the above + Woe face	→	*n/a*	*n/a*
Face not visible	→	Face not found	Not found

Throughout this work, we compared the outputs of manual and automated coding methods, and examined the potential impact of parent gender on model accuracy by analysing separate and combined datasets for fathers and mothers. This comprehensive analysis sought to uncover the application of AFC in capturing and interpreting parent facial expressions within naturalistic contexts, whilst also exploring any gender-specific nuances or variations that may influence the accuracy of the models. Whilst acknowledging the significance of exploring emotional facial expressions and parental gender, our primary focus lies in comparing automated and manual coding and investigating the associations between these two approaches. By prioritising these aspects, we aim to provide valuable insights into the methodological implications of automated facial coding and its associations with human coding, shedding light on the strengths, limitations, and potential applications of AFC in analysing facial expressions.

## 2. Materials and methods

### 2.1. Data

This work used data from the Avon Longitudinal Study of Parents and Children (ALSPAC). ALSPAC is an ongoing longitudinal cohort study based in Bristol, United Kingdom. The original cohort—referred to as ALSPAC-G0—was recruited via 14,541 pregnancies with expected delivery dates between 1 April 1991 and 31 December 1992. The children born to the ALSPAC-G0 cohort are referred to as ALSPAC-G1, and the children born to the ALSPAC-G1 cohort (in recent years) are referred to as ALSPAC-G2. Full ALSPAC cohort demographics have been provided elsewhere (Boyd et al., 2013; Fraser et al., 2013; Lawlor et al., 2019; Northstone et al., 2019).

This work uses videos of parents from ALSPAC-G1. Parents (regardless of gender) were recruited though research clinics at the University of Bristol, which invited parents to complete several assessments when their infant turned 6 months old. There were no selection criteria to take part in the study, other than being either part of the original ALSPAC cohort, or a partner of an original ALSPAC participant.

The fathers in this work had a mean age of 31.3 years (*SD* = 5.5), and their infants had a mean age of 32.6 weeks (*SD* = 5.9). Eight infants in father-infant dyads were male, and five infants were female. The mothers in this work had a mean age of 29.9 years (*SD* = 1.1), and their infants had a mean age of 49.8 weeks (*SD* = 11.9). In the mother-infant dyads, five infants were male, and nine infants were female. All mothers identified their gender as female, and all fathers identified as male.

The study website contains details of all ALSPAC data that are available through a fully searchable data dictionary and variable search tool. ^1^ ALSPAC data are collected and managed using Research Electronic Data Capture (REDCap) electronic data capture tools hosted at the University of Bristol (Harris et al., 2009). REDCap is a secure web-based platform designed to support data capture for research studies. Ethical approval for the study was obtained from the ALSPAC Ethics and Law Committee and the Local Research Ethics Committees. Informed consent for the use of data collected via questionnaires and clinics was obtained from participants following the recommendations of the ALSPAC Ethics and Law Committee at the time.

#### 2.1.1. Video recording procedures

The parents were provided with fully-charged wearable headcams and asked to wear them during interactions with their child at home. They were also given an information sheet explaining how to wear and use the cameras. Due to the videos being taken at home, it was possible that siblings/other caregivers/pets were present during the interactions. The videos were collected between 2019 and 2022; each has a frame rate of 30, and a resolution of 1,280 × 720.

The videos include a combination of different interaction types: feeding (infant eats a meal), free play (parent/infant engages in an unstructured play session), and stacking (parent/infant plays with a given stacking toy). We used 36 videos of fathers in total, including: 24 feeding, 10 free play, and two combined interactions (included both feeding and free play). For the mothers, we used 29 videos in total, including: 19 feeding, six free play, and four stacking task interactions. These videos come from 13 individual fathers and 14 individual mothers, as many parents provided multiple separate videos. Due to variation in video length, it was often the case that one parent provided multiple videos equating to the length of a single video from another parent. For this reason, we decided not to exclude second (or more) videos from a single parent. Overall, the mean video length was 433.0 s (*SD* = 173.0); the mean length of the father videos was 482.8 s (*SD* = 192.7), whilst the mean length of the mother videos was 383.1 s (*SD* = 124.4).

#### 2.1.2. Manual coding

All videos were manually coded using Noldus Observer 15.0 (Noldus, 1991) at a slowed speed of 0.2 s (i.e., the video was slowed to 0.2 s in order to code expressions on an events-based approach). A speed of 0.2 s was chosen as it provided a balance between capturing detailed behavioural information and managing the time and resource constraints associated with the coding process in our study. Coding was carried out according to an existing coding scheme (Costantini et al., 2021); whilst this coding scheme includes a wide range of behaviours, the work here only included facial expressions. Analyses incorporating the other behaviours can be found elsewhere (Campbell et al., 2022; Burgess et al., 2023).

We used the following manually-coded facial expressions: Smile, Positive, Neutral/Alert, Negative, Surprise, Mock Surprise, Woe Face, Disgust, None of the Above, and Face not visible. These expressions are exhaustive and mutually exclusive, such that one expression is coded for every point in time. It should be noted that None of the Above is used to indicate any meaningful expression that does not fit into any other domain (e.g., yawning, sneezing).

Four researchers were involved in the coding process (RB, IC, LR, and MS). All were independently trained in using the coding scheme. Initial coding of all videos was performed by one researcher (RB). For the fathers, two additional researchers were recruited for double coding (LR and MS). Seven randomly selected videos were selected for double coding, with one researcher coding four videos, and one researcher coding three. This equated to 22.4% of the father data. For the mothers, one additional researcher was recruited for double coding (IC). Six randomly selected videos were selected for double coding, equating to 15.7% of the mother data. In total, across both sets of parents, 19.0% of data was double coded.

We used the *index of concordance* to measure inter-coder agreement. The index of concordance is calculated by the total agreement for a behaviour (i.e., the duration that an expression is coded as present/not present by both coders) divided by the total duration of the interaction. This is expressed as a value between 0 (no agreement) and 1 (total agreement). For the fathers, an index of concordance of 0.93 (*SD* = 0.07) was achieved with the first double coder, and 0.91 (*SD* = 0.07) was achieved with the second coder. For the mothers, an index of concordance of 0.87 (*SD* = 0.02) was achieved between the two coders. Inter-coder agreement by facial expression and parent gender are provided in Table 2. This analysis excludes expressions that occurred for less than 1% of time across all interactions (i.e., Woe face, Disgust, and Surprise).

**Table 2:** Mean (SD) inter-coder agreement by facial expression and parent gender.

	Neutral	Positive	Smile	Negative	Mock surprise	None of the above
Fathers	0.89 (0.09)	0.89 (0.09)	0.96 (0.03)	0.98 (0.00)	0.98 (0.02)	0.94 (0.05)
Mothers	0.89 (0.04)	0.80 (0.05)	0.88 (0.07)	0.91 (0.00)	0.90 (0.04)	0.82 (0.05)
All	0.89	0.84	0.92	0.95	0.94	0.88

**Figure 1:**
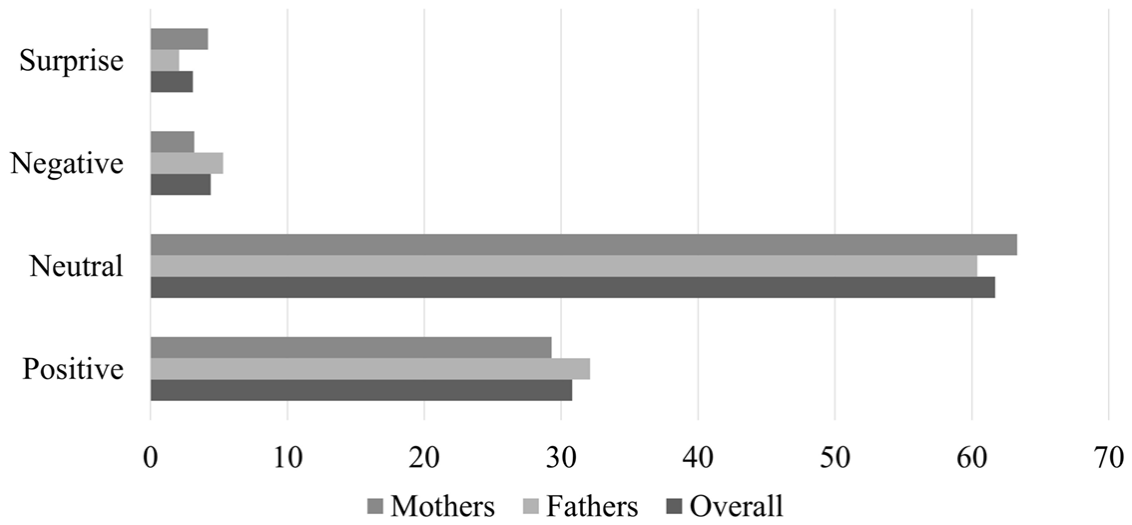
Proportion of each expression category in the data.

As outlined in the introduction, due to the discrepancies between the expressions in the automated and manual coding methods, we used mappings to establish relationships between the two expression sets: Positive, Neutral, Negative, and Surprise (see Table 1). These mappings were specifically created for the contingency analyses and to serve as target variables in the logistic regressions. Once the expressions had been aggregated into these four new categories, we calculated the proportions of each category within the datasets. This analysis aimed to highlight expressions with low prevalence and identify any imbalances between the father and mother datasets. The findings of this analysis are presented in Figure 1, illustrating the proportions of each manually coded category within the full dataset, as well as the father and mother datasets.

In each case, Neutral is the most prevalent expression, accounting for around 60% of the data. This is followed by Positive, which represents around 30%. Negative and Surprise both represent less than 5% of the data, with Negative being more common in fathers, and Surprise being more common in mothers.

#### 2.1.3. Automated facial coding using FaceReader

All videos were processed using Noldus FaceReader 8.0 (Noldus, 2014). The software uses deep learning to locate faces in an image, and eye tracking to identify the face rotation. An artificial mesh is placed over 468 key points on the face, describing the position of features and muscles. Principal component analysis condenses these points into a single vector representation, describing the main facial features. Finally, expression classification takes place using a neural network which has been trained on more than 20,000 manually-coded images of faces (Gudi et al., 2015). A more detailed explanation of this process can be found elsewhere (Loijens et al., 2020).

Given a face, FaceReader calculates an *expression intensity:* a single value between [0, 1] describing the strength of an expression. An intensity of 0 indicates the expression is not present, and an intensity of 1 indicates an expression is entirely present. FaceReader provides an intensity for each of the eight expressions simultaneously, with each value independent of one another. FaceReader processes videos frame-by-frame; our videos had a frame rate of 30, meaning FaceReader analysed 30 frames per second.

### 2.2. Methods

#### 2.2.1. Data pre-processing

In terms of pre-processing, we first removed all data where an additional person (e.g., a second caregiver, a sibling) was present in the video. This might occur when, for example, a second caregiver was bringing food or play items or walking in the background. We removed these data as FaceReader would sometimes classify the facial expression of the second person instead of the participant. After removing these data, the amount of viable coded data for fathers reduced from 17,368 to 15,420 s, and the amount of viable coded data for mothers reduced from 11,509 to 10,614 s.

We also normalised the expression intensities (to sum to 1) to ensure that there was always a dominant expression within the FaceReader output. This was necessary in order to remain consistent with the manual coding, which always determines one dominant facial expression at any given time. All analyses were carried out using Python 3.0 (Van Rossum and Drake, 2009).

#### 2.2.2. Data analysis procedures

To address (A1), we used contingency analysis to quantify the amount of time that the parent’s face was found by both the manual coder and the automated facial coding, i.e., the facial detection rate. This involved a frame-by-frame analysis, categorising each frame dependent on whether the human and the software successfully detected a face.

To address (A2) we used a second contingency analysis, followed by multiple binary logistic regression models. This second contingency analysis quantified the agreement on the four descriptive expression categories—Positive, Neutral, Negative and Surprise—as coded manually and automatically. Additionally, we used logistic regression models to establish whether the automated facial coding was predictive of the manual facial coding. Here, our models used the eight normalised FaceReader expression intensities to predict the four manually coded categories (which were classified as a 1 if the expression was present, and as a 0 if not).

To address (A3), we compared the performance of combined and gender specific logistic regression models. The logistic regression analyses were therefore carried out in three parts—fathers only, mothers only and combined (using both mothers and fathers)—facilitating an analysis of gender discrepancies within the models.

Across our study, we employed a leave-one-out cross-validation approach to train, fit, and evaluate logistic regression models for each manually coded expression category. The process involved several steps, and this whole process was repeated for each of the manually coded categories: Positive, Neutral, Negative and Surprise. The steps of this process are outlined below:

**Step 1: data preparation and train-test splitting.** We prepared the dataset by separating it into train-test sets, and then into predictors and target variables. For each iteration of cross-validation, we excluded one individual as the test set whilst using the remaining individuals as the training data. This ensured that each individual had an opportunity to serve as the test set. Next, we specified the input variables—the eight FaceReader expression intensities—and the target variable—the manually coded expression. The target variable was coded as a 1 if the expression was present, and a 0 if the expression was not. Separate logistic regression models were developed, trained, and optimised for each expression category. Note that, in the combined models, we included gender as a binary input variable alongside the expression intensities.

**Step 2: model training.** Logistic regression models were trained using the scikit-learn library in Python, specifically the *LogisticRegression* classifier. This classifier is popular for binary classification and supports multiple solvers, including the efficient LBFGS solver used in this study. The LBFGS solver optimises logistic regression models by finding the parameter values that maximise the likelihood of the observed data (Fletcher, 2000). The models were trained with a maximum of 10,000 iterations, and the class weights were balanced to account for the imbalance in expression frequencies. This weighting strategy helped ensure that the models were trained effectively despite variations in expression frequencies (see Figure 1).

**Step 3: model fitting.** The trained models were fitted to the data, automatically adjusting the model parameters to best fit the training data. This step allowed the models to fine-tune their performance based on the training data, improving their ability to accurately predict the presence or absence of the target expression category.

**Step 4: model evaluation.** To evaluate the model’s performance, various metrics—accuracy, specificity, and sensitivity—were calculated using the true labels from the test data and the predicted labels (from applying the model to the test data). Accuracy indicates the proportion of correct predictions for both classes 1 (the expression is present) and 0 (the expression is not present). Sensitivity indicates the model’s ability to predict a true positive; if sensitivity is high, the model correctly classifies existing expressions as present, rather than not present. Finally, specificity indicates the model’s ability to predict a true negative; if specificity is high, the model correctly classes absent expressions as not present, rather than present.

To provide a comprehensive understanding of the performance estimates and quantify the associated uncertainty, we calculated the mean value and 95% lower and upper bounds for each metric. These bounds captured the range of likely values for the metrics and offered insights into the precision and reliability of the model’s performance. They added a measure of confidence to the model’s performance evaluation and allowed us to better interpret and compare the results across different models and expression categories.

**Step 5: repeat.** We defined a new train and test set, using a different participant as the test data, then repeated steps 2-4 until all participants had formed the test set at one stage.

In summary, our approach involved preparing the data, separating it into training and test sets, specifying the input and target variables, training the models, fitting the models to the data, and evaluating the model’s performance using cross-validation. This process was repeated using each participant as the test set, and using each manually coded expression as the target. The inclusion of a leave-one-out cross-validation strategy allowed us to assess the performance of the models for each manually coded expression category. The collected metrics, including accuracy, specificity, and sensitivity, were further summarised using mean values and 95% lower and upper bounds, providing a comprehensive understanding of the models’ performance.

## 3. Results

Our results are split into two sections. Section 3.1 presents our contingency analyses: first to quantify facial detection, second to quantify the agreement between AFC and manual coding. Section 3.2 presents the logistic regression models used to evaluate the relationship between human and manual coding, first as with all data, and then separated by gender.

### 3.1. Contingency analyses

Here, we discuss the findings from our contingency analyses. This section is split into two parts: (1) quantifying facial detection rate, and (2) quantifying the link between manual and automated facial coding.

#### 3.1.1. Quantifying facial detection rate

To address (A1), we used contingency analysis to quantify how frequently both the human and the software classified a face. Our results are provided in Table 3. The following discussion assumes the manually coded expressions to be correct.

**Table 3:** Face found vs. not found by FaceReader and the manual coder.

Parent	Manual	FaceReader (%)	
		Face found	Face not found
Fathers	Face found	10.71	31.84
	Face not found	0.47	57.00
Mothers	Face found	12.79	39.18
	Face not found	0.26	47.77

The contingency analysis included 26,034 s of data (fathers *n =* 15,420, mothers *n =* 10,614). For the fathers, the researcher found a face 42.55% of the time, whilst FaceReader found a face 11.18% of the time (from Table 3: manual face found = 10.71 + 31.84, FaceReader face found = 10.71 + 0.47). For the mothers, the researcher found a face 51.97% of the time, whilst FaceReader found a face 13.05% of the time.

We can also interpret the percentage of time where FaceReader found a face, but the manual coder did not (0.47% for fathers, 0.26% for mothers). This is a rare occurrence, and indicates where FaceReader mistakenly classifies another object as the parent face (e.g., a face on a poster or clothing).

FaceReader facial detection rate can be extracted by looking at—of the frames where a face was present (and coded as present by the human coder)—how frequently FaceReader successfully found a face. To calculate this, we divided the percentage of face found by both (manual and FaceReader) by the total manual face found. This gives a facial detection rate of 25.17% for the fathers and 24.61% for the mothers (or 24.89% overall). The ‘successfully’ classified observations comprise the data used in the logistic regression models. Explicitly, observations were only included if both FaceReader and the manual coder identified a face. For the fathers, this reduced the dataset to 49,532 frames (around 1,651 s), and for the mothers, this reduced the dataset to 40,721 frames (1,357 s).

#### 3.1.2. Quantifying the relationship between human and automated facial coding

To address (A2), we used a contingency table to quantify the agreement between four expressions categories—Positive, Negative, Neutral and Surprise—as coded manually and automatically. The results of this analysis are shown in Table 4.

**Table 4:** Confusion matrix of manually and automatically coded expressions (%).

Manual	AFC			
	Positive	Neutral	Negative	Surprise
Positive	20.40	9.07	0.73	0.62
Neutral	56.76	0.78	2.86	1.32
Negative	3.94	0.05	0.28	0.08
Surprise	2.17	0.16	0.12	0.64

First, both manual coding and AFC agreed that faces were positive 20.40% of the time overall, indicating a moderate level of agreement in identifying positive expressions. However, there is a significant discrepancy between the two methods in categorising faces as Neutral. Manual coding labelled faces as Neutral, whilst AFC classified the same faces as Positive in 56.76% of cases. This suggests that AFC tends to be biassed towards categorising faces as Positive, whilst manual coding leans more towards Neutral. Additionally, AFC often identified faces as Neutral whilst manual coding labelled them as Positive (9.07% of the time).

There were instances where AFC categorised faces as Negative whilst manual coding classified them as Neutral (2.86%). In contrast, when manual coding categorised faces as Negative, AFC often believed the same faces to be Positive (3.94%). These findings indicate discrepancies in the interpretation of negative expressions between the two methods. Similarly, when AFC categorised faces as Surprise, manual coding classified them as Positive (2.17%), yet when manual coding categorised faces as Surprise, AFC classified them as Neutral (1.32%). These results suggest differences in detecting and classifying surprise expressions between manual coding and AFC.

### 3.2. Logistic regression analyses

This section reports the findings from our logistic regression analyses, with results split into two parts: (1) the combined models, and (2) the gender-specific models. Here we address both aims (A2) and (A3). For (A2), this is done by continuing to explore the relationship between manual and automated facial coding, using logistic regression on the automated outputs in order to predict the manual coding. For (A3), this is done by carrying out distinct logistic regression analyses for both combined and gender specific datasets.

#### 3.2.1. Combined models

This analysis used a combined dataset comprised of both father and mother data, employing logistic regression models to predict the manually coded expressions using FaceReader expression intensities and gender as predictor variables. First, we provide some summary statistics from FaceReader, detailing the mean (SD) expression intensities (across all time points) for the mothers and fathers separately (see Table 5).

**Table 5:** Mean (SD) expression intensities, as coded by FaceReader, separated by gender.

Expression	Mean intensity (SD)	
	Mothers	Fathers
Happy	0.85 (0.21)	0.56 (0.22)
Sad	0.11 (0.16)	0.09 (0.12)
Neutral	0.15 (0.25)	0.14 (0.21)
Angry	0.08 (0.12)	0.05 (0.08)
Scared	0.04 (0.09)	0.01 (0.03)
Surprised	0.17 (0.21)	0.07 (0.09)
Disgusted	0.06 (0.11)	0.04 (0.08)
Contempt	0.01 (0.02)	0.01 (0.02)

Table 5 shows that the mean expression intensity was almost always higher in mothers than fathers. The largest differences in intensities between genders were found for Happy (mothers = 0.85, fathers = 0.56) and Surprised (mothers = 0.17, fathers = 0.07). The remaining expressions showed similar intensities (and standard deviations) between genders. Notably, the standard deviation for Surprised was particularly large in the mothers compared to the fathers.

We then used logistic regression models to quantify the relationship between manual and automated coding. Gender was included as an additional, binary input variable within the models—represented by either a 0 (mothers) or a 1 (fathers). By interpreting the direction of this coefficient (negative or positive), we can assess which parent gender is most important for predicting a given expression. We used the combined dataset for this analysis, comprising both father and mother data, fitting separate binary logistic regression models for each expression category (Positive, Neutral, Negative and Surprise). The leave-one-out, cross validation approach for model evaluation was outlined in Section 2.2.2. The resulting predictive performance measures—accuracy, sensitivity and specificity—are provided in Table 6, along with the value of the gender coefficient.

**Table 6:** Accuracy, sensitivity and specificity metrics for cross-validated, combined models.

	Accuracy		Specificity		Sensitivity		Coef^**^	
	Mean	Range^*^	Mean	Range	Mean	Range	Mean	Range
Positive (*n* = 26,706)	0.76	0.70–0.81	0.60	0.46–0.73	0.83	0.76–0.91	−0.06	−0.14–0.03
Negative (*n* = 3,776)	0.54	0.40–0.67	0.51	0.30–0.72	0.54	0.39–0.68	0.67	0.58–0.78
Neutral (*n* = 50,682)	0.68	0.62–0.74	0.76	0.66–0.86	0.57	0.44–0.70	−0.03	−0.10–0.04
Surprise (*n* = 2,671)	0.80	0.69–0.92	0.34	0.15–0.53	0.81	0.69–0.93	1.42	0.93–1.54

^*^Range indicates the 95% upper and lower bounds on the given performance metric (e.g., accuracy). ^**^Coef refers to the value of the ‘gender’ coefficient in the LR models.

The results in Table 6 indicate variations in performance across different facial expression categories. Overall, the Surprise models showed the highest accuracy with a mean of 0.80 (*range* = 0.69–0.92), indicating strong overall prediction performance for both the presence and absence of Surprise expressions. Additionally, the mean sensitivity for Surprise was notable at 0.81 (*range* = 0.69–0.93), indicating the model’s ability to correctly identify existing Surprise expressions. However, the mean specificity for Surprise was the lowest amongst all expressions at 0.34 (*range* = 0.15–0.53), suggesting challenges in accurately classifying absent Surprise expressions. The Positive models exhibited a high mean accuracy of 0.76 (*range* = 0.70–0.81), indicating a relatively high proportion of correct predictions for both the presence and absence of Positive expressions. They also demonstrated the highest sensitivity amongst all the models, with a mean of 0.83 (*range* = 0.76–0.91), meaning they effectively captured and correctly classified existing Positive expressions as present. Additionally, the models showed moderate specificity, with a mean of 0.60 (*range* = 0.46–0.73), suggesting a reasonable ability to accurately classify absent Positive expressions as not present.

The Negative models exhibited a relatively lower accuracy compared to other categories (*mean* = 0.54, *range* = 0.40–0.67), a modest sensitivity (*mean* = 0.54, *range* = 0.39–0.68), and a moderate specificity (*mean* = 0.51, *range* = 0.30–0.72). Together, these results indicate that the Negative models demonstrated greater accuracy in correctly identifying non-Negative expressions compared to identifying Negative expressions, suggesting a tendency for more accurate detection of expressions other than Negative. The Neutral models achieved a relatively high accuracy (*mean* = 0.68, *range* = 0.62–0.74), a moderate sensitivity (*mean* = 0.57, *range* = 0.44–0.70), and a high specificity (*mean* = 0.76, *range* = 0.66–0.86). These findings suggest that the models had a reasonable ability to correctly identify non-Neutral expressions compared to identifying Neutral expressions. They demonstrated a higher tendency to accurately classify expressions as non-Neutral, indicating a relatively stronger capability in detecting and distinguishing other types of expressions.

Table 6 also reveals insights into the links between gender and facial expressions. Mothers’ faces, as automatically coded, play a larger role than fathers’ faces in predicting Positive expressions, as indicated by the negative coefficient of −0.06 (with variability as shown by the *range* = −0.14 to 0.03). Similarly, mothers’ faces are more important for predicting Neutral expressions (*coef* = −0.03, *range* = −0.10 to 0.04). Conversely, fathers’ faces are more important for predicting Negative (*coef* = 0.67, *range* = 0.58 to 0.78) and Surprise expressions (*coef* = 1.42, *range* = 0.93 to 1.54), as indicated by the positive coefficients.

#### 3.2.2. Gender-specific models

This section carries out identical logistic regression analyses, but with two separate datasets: one of mother data and one of father data (gender is no longer included as a predictor variable). For these two gender specific datasets, we fit separate logistic regression models for each manually coded category, as outlined in Section 2.2.2. Following a cross-validated approach, mean predictive performance measures for these models are shown in Table 7.

**Table 7:** Accuracy, sensitivity and specificity metrics for cross-validated, gender-specific models.

	Accuracy		Specificity		Sensitivity	
	Mean	CI (95%)	Mean	CI (95%)	Mean	CI (95%)
Fathers						
Positive (*n* = 15,152)	0.74	0.71–0.78	0.58	0.46–0.67	0.81	0.75–0.88
Negative (*n* = 2,507)	0.58	0.46–0.70	0.42	0.25–0.59	0.59	0.45–0.73
Neutral (*n =* 25,713)	0.70	0.65–0.75	0.79	0.71–0.88	0.54	0.44–0.65
Surprise (*n* = 1,003)	0.84	0.76–0.91	0.38	0.18–0.58	0.85	0.77–0.92
Mothers						
Positive (*n* = 11,554)	0.74	0.69–0.84	0.67	0.52–0.82	0.81	0.72–0.91
Negative (*n* = 1,269)	0.53	0.43–0.63	0.48	0.27–0.68	0.52	0.41–0.63
Neutral (*n =* 24,969)	0.66	0.58–0.73	0.69	0.56–0.81	0.64	0.49–0.79
Surprise (*n* = 1,668)	0.73	0.61–0.85	0.35	0.15–0.55	0.74	0.61–0.86

Table 7 shows that for fathers, the mean accuracy ranged from 0.58 to 0.84 across the expression categories. The highest accuracy was observed for the Surprise models—indicating that models performed well in predicting the presence or absence of surprise in fathers—whilst the lowest accuracy was observed for the Negative models. Mean specificity ranged from 0.38 to 0.79, suggesting varying success in correctly identifying non-expressions. Notably, the Surprise category had the lowest specificity, indicating that models particularly struggled to classify the absence of Surprise in fathers. Mean sensitivity ranged from 0.54 to 0.85, with the Surprise category displaying the highest value and Neutral displaying the lowest. This suggests that models were able to correctly classify when Surprise was present, but could not accurately predict the presence of Neutral.

Shifting to mothers, the mean accuracy ranged from 0.53 to 0.74 across the expression categories. Similar to fathers, high accuracy for mothers was observed in the Surprise and Positive models. Mean specificities were low to moderate, ranging from 0.35 to 0.69, indicating that models struggled to identify absence of expressions. The highest specificity was observed in the Neutral category, and the lowest was for Surprise. Mean sensitivity values for mothers ranged from 0.52 to 0.81, with the highest observed in the Positive models, and the lowest for the Negative models. This suggests that models performed well at classifying when Positive was present, but not so well at classifying presence of Negative.

The models for fathers and mother expressions exhibited differences in their accuracy, specificity and sensitivity across the categories, with the father models performing slightly better overall. Both genders showed high accuracy in the Surprise and Positive models, and showed considerably lower performance in the Negative models. The models for Neutral were most specific in each case, whilst Surprise was least specific. Finally, there were differences in sensitivity, as Surprise models were most sensitive for fathers, whilst Positive were the highest for mothers.

## 4. Discussion

### 4.1. Summary of results

Manual facial coding can be laborious, time consuming, and subject to human biases. Automated facial coding (AFC) offers a rapid and objective alternative. Previous literature has evaluated applications of AFC across various contexts, however, most work has focused on videos taken by stationary cameras, in heavily controlled environments, with good lighting and homogenous backgrounds (Benţa et al., 2009; Danner et al., 2014; Brodny et al., 2016). Fewer studies have investigated the use of AFC for more dynamic and naturalistic observations, which is important to investigate as it allows for a better understanding of spontaneous emotional expressions in real-world contexts, capturing the intricacies of human interactions and reactions (Lee et al., 2017). Additionally, whilst some works have used AFC to analyse parent facial expressions (Karreman and Riem, 2020; Lyakso et al., 2021), there remains scope for deeper investigations, including those evaluating differences in gender.

Thus, our study was carried out with three primary aims: (A1) assess the facial detection rate of the software, (A2) quantify the agreement between automated and manual facial coding: by assessing the concordance between the two methods, and using the automated outputs to predict the manual coding, and (A3) investigate the influence of parent gender on the relationship between manual and automated facial coding, by analysing combined datasets as well as separate datasets for fathers and mothers. To address these aims, we compared manual and automated facial coding for 65 videos of parents (*n =* 36 fathers, *n* = 29 mothers), taken using wearable headcams during interactions at home. We used a total of 26,034 s of data after pre-processing (fathers *n =* 15,420, mothers *n =* 10,614). The videos were coded both manually—using the MHINT coding scheme (Costantini et al., 2021)—and automatically—using the FaceReader software (Noldus, 2014). The first stage of our analyses involved using contingency tables to quantify automated facial detection rate, as well as the agreement between manual and automated coding. The second stage used combined and gender specific logistic regression models to further evaluate this relationship, and estimate the influence of gender on the models.

Section 3.1.1 showed that automated facial coding detected a face around 25% of the time that the human coder did. Similarly, the contingency analysis in Section 3.1.2 found that whilst there was a large overlap in classifying expressions as Positive, automated facial coding showed a large bias towards classifying faces as Positive, where manual coding denoted them as Neutral. There were additional discrepancies between the other expression categories.

Using the full dataset, Table 6 revealed variations in model performance across different facial expression categories. Overall, the Surprise and Positive models exhibited the highest accuracies and sensitivities, indicating strong predictive capabilities for these expressions. The Negative models were low poor performing, whilst the Neutral models achieved reasonable accuracy and high specificity. Regarding gender differences, the coefficient values highlighted that mothers’ faces play a slightly more important role than fathers’ faces in predicting Positive and Neutral expressions, whilst fathers’ faces are more influential in predicting Negative and Surprise expressions.

The father specific models showed the highest accuracy in the Surprise models, whilst the mother specific models showed highest accuracy was observed in the Positive models. Both had lowest accuracy in the Negative models, and the gender specific models varied in their ability to accurately identify the presence or absence of each expression.

### 4.2. Failures in facial detection

Whilst it is important that we begin to trial automated facial coding in more naturalistic environments (Matlovic et al., 2016), this is very difficult in practise (Gómez Jáuregui and Martin, 2013). Across many of the videos in our work, FaceReader struggled to locate and classify faces, as indicated by the low detection rates of around 25%. A detection rate of 25% is not surprising, as real-world conditions are dynamic and complex—for example, on account of varied movements and surroundings—meaning that automated facial recognition is disadvantaged (Gómez Jáuregui and Martin, 2013). We thus consider the low FaceReader success to be indicative of the high ecological validity of our videos. As opposed to previous studies—where data were excluded based on FaceReader’s ability to analyse it (Weth et al., 2015)—we retained these data for the purpose of validating the software for real-life conditions.

**Figure 2:**
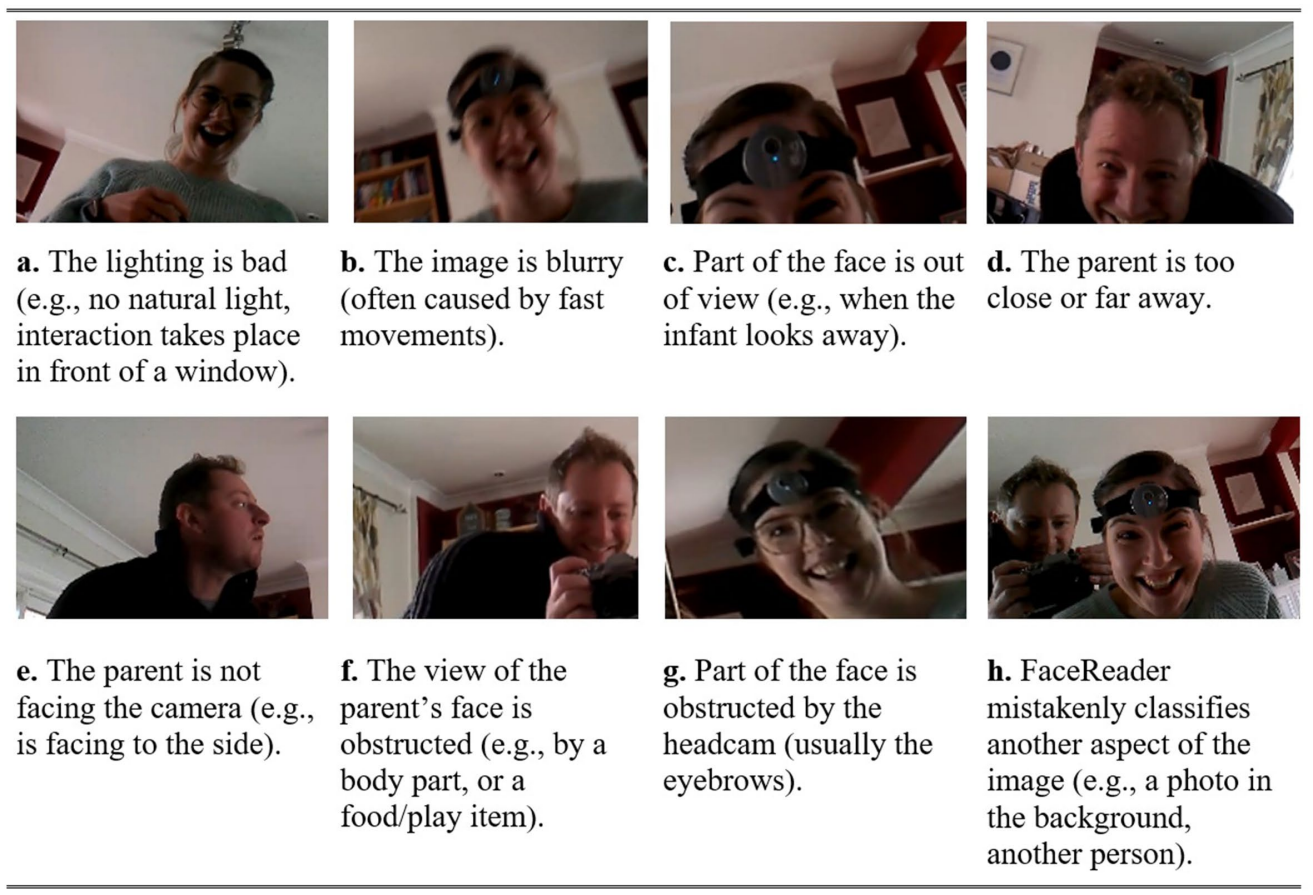
Video snapshots where FaceReader fails to detect a face, for reasons as specified: **(A)** poor lighting conditions, **(B)** blurry images, **(C)** part of face out of view, **(D)** distance from headcam, **(E)** parent facing away, **(F)** face obstructed by object, **(G)** face obstructed by headcam, and **(H)** mistaken classification.

By looking back through our videos, we have identified potential reasons for the failures in facial detection; an overview of this is provided in Figure 2. This figure shows eight images taken using the headcams, with an accompanying explanation to describe why automated facial detection would not work well within the image. These reasons are: poor lighting conditions (a), blurry images (b), incomplete view of the face (c, d, and e), obstructed view of the face (f and g), or mistaken classification (h). For confidentiality reasons, we could not share photos of the participants used in this work. However, Figure 2 contains comparable images from a mother and father who consented to have their data shared.

Previous work has highlighted that dark lighting (Loijens et al., 2020) or artificial illumination (Gómez Jáuregui and Martin, 2013) negatively impacts automated facial detection. In our videos, many interactions took place in artificially lit rooms with no natural light, in front of bright windows, or in rooms with no lighting at all (depending on the time of day). These factors impacted the illumination on parents’ faces, resulting in reduced capability of the automated facial coding software to accurately identify crucial facial features necessary for expression determination. In some cases, the software faced challenges in detecting faces altogether (see Figure 2A).

The type of interactions used in this work—feeding (*n =* 43), free play (*n =* 16), stacking (*n =* 4) and combination (*n =* 2)—meant that our videos contained dynamic, fast body movements and variation between positions (e.g., sitting and lying on the floor during play). These rapid movements sometimes led to blurry images (see Figure 2B), causing the automated facial coding software to struggle to identify the facial features required for expression classification.

Additionally, these dynamic interactions often meant that the face was out of view of the camera (see Figure 2C). This happened when the infant looked away, the parent moved too close or far away (see Figure 2D), and when the parent faced away from the infant (see Figure 2E). In feeding interactions, for example, the parent might be sat at the table or next to the infant, leading to sideways or otherwise indirect views of the face. Whilst humans can identify facial expressions from an angled (or partially obscured) view, FaceReader is not able to distinguish faces beyond a 40-degree tilt (Loijens et al., 2020). As another example, consider free play or stacking tasks; if the infant threw a toy out of view, the parent would move further away to retrieve it. This distance would decrease the software’s ability to detect facial features. Although these factors are indicative of natural interactions, they led to a decrease in successful automated facial detection.

Figure 2F demonstrates an obstruction of the face. Previous studies indicate that glasses, facial hair, hats, or other objects can hinder automated facial detection (Gómez Jáuregui and Martin, 2013; Booijink, 2017; Loijens et al., 2020). Amongst our participants, a small subset of six fathers sported facial hair, whilst a few fathers and seven mothers were observed wearing glasses. It is also noteworthy that several parents had hairstyles featuring fringes that partially obstructed their eyebrows. Additionally, during the interactions, objects such as cutlery or toys were frequently raised in front of the parent’s or infant’s face, resulting in partial or complete obstruction of the facial region. In such cases, automated facial detection is at a disadvantage compared to human facial detection.

Figure 2G shows how the headcam itself could also serve as an obstruction. This often occurred when the headcam was placed too low on the parents’ face, covering the eyebrows. Without the ability to locate the eyebrows, the automated facial coding software would struggle to map the key points on the face and classify an expression. In this case, the human coder is likely to still be able to classify an expression based on other visible facial features (e.g., eyes, mouth). Previous work using headcams to capture dyadic interactions allowed the researchers to adjust the cameras on the participants themselves (Park et al., 2020), to ensure that they were well fitted and angled correctly. This was not possible in our work, as the parents fitted both their own and their infants’ headcams, meaning that errors in placement were more likely to occur.

Table 3 highlighted the amount of time where automated facial detection found a face within the image, but the human coder did not (fathers = 0.47%, mothers = 0.26%). These percentages describe instances where the software misclassified another object or person as a face (see Figure 2H). As these values are low, this was not a common occurrence. We removed frames with additional people present during pre-processing, such that software misclassifications in our data were more likely caused by items with faces (or shapes resembling faces) on (e.g., clothing, photos or stuffed animals). The negative impact of complex backgrounds on automated facial detection has been reported previously (Gómez Jáuregui and Martin, 2013).

### 4.3. Implications for automated facial coding in naturalistic environments

Our research has highlighted the importance of incorporating naturalistic observations in training AFC models, addressing limitations associated with relying solely on standardised and controlled environments. Whilst FaceReader, a widely used AFC software (Den Uyl and Van Kuilenburg, 2005; Noldus, 2014), has shown efficacy in specific conditions (Höfling et al., 2021), challenges persist in accurately distinguishing between neutral and unpleasant stimuli and effectively handling non-standardised expressions (Höfling et al., 2020; Küntzler et al., 2021). Nevertheless, AFC has demonstrated promise beyond academic research, particularly in predicting self-reported emotions and providing valuable insights in marketing research, where it captures non-verbal aspects (Höfling and Alpers, 2023).

Unlike previous studies that primarily used laptop webcams (Danner et al., 2014; Booijink, 2017; Talen and den Uyl, 2022), which fail to capture dynamic and complex real-life interactions, our study employed wearable headcams, offering a first-person perspective that aligns with naturalistic observations. Through evaluating scenarios where automated facial detection and coding faced challenges, we provide recommendations to optimise future studies’ success. These recommendations [1]–[6], previously introduced in (Burgess et al., 2022), are reiterated here with two additional points and further details.

1. Provide participants with clear guidance on how to properly fit the headcam, ensuring it sits above the eyebrows and points in the appropriate direction to capture the partner’s face.
2. Advise participants on optimal lighting conditions, preferably recording in natural light. Alternatively, explore options such as FaceReader’s new infrared video analysis feature (Noldus Information Technology, 2023) to improve accuracy in videos with poor lighting.
3. Encourage participants to minimise facial occlusions by avoiding glasses, hats, and keeping hair away from the face. However, recognise that certain behaviours involving objects in front of the face should not be discouraged to maintain authenticity.
4. In some contexts (e.g., feeding), participants may be informed of specific body positions to maintain, whilst in others, maintaining the dynamic nature of naturalistic interactions is essential (e.g., play).
5. Participants should be advised against wearing clothing with faces displayed on them and recording in areas with complex backgrounds. Alternatively, future research could explore post-production techniques to reduce mistaken classification, such as blurring or obscuring backgrounds.
6. Supporting the development of more powerful compact cameras that are robust against rapid head movements can further enhance the performance of AFC models in naturalistic settings.
7. Advise participants to maintain a comfortable distance from the camera to avoid excessive close-ups or wide-angle shots that may affect the accuracy of facial expression analysis. Providing specific guidance on the optimal distance can help standardise the data collection process.
8. Conduct a pilot study to help familiarise participants with the headcam and its proper usage, allowing them to adjust and become more comfortable with the equipment. Additionally, a pilot study can provide an opportunity to fine-tune the recording setup, lighting conditions, and other variables to optimise the performance of the AFC models in naturalistic settings.

Implementing these recommendations can help researchers optimise the success of automated facial detection and coding on videos of naturalistic observations. However, it may be necessary to combine automated facial coding with manual coding or other complementary techniques at this stage, such as: using human raters for specific facial expressions that automated systems struggle to accurately detect, or employing machine learning algorithms to improve the performance of automated coding in challenging scenarios. Another approach could be to use FaceReader to automatically code a portion of the data, approximately 25% based on observed success rates, and supplement the remaining data with manual coding. Even with a success rate of 25%, automated coding techniques can be valuable, especially when dealing with large datasets or time constraints.

Table 4 demonstrated both areas of agreement and notable disagreements between manual coding and AFC in classifying naturalistic facial expressions. Whilst there was some agreement in identifying positive expressions, substantial differences arose in categorising faces as Neutral, Negative and Surprise. These findings suggest the presence of biases in the classifications of both methods and highlight the importance of considering human judgement or complementary techniques alongside automated systems like AFC for a comprehensive analysis of facial expressions.

Overall, the limitations and challenges in applying AFC to real-life recordings are evident. We have provided practical recommendations for optimising AFC in naturalistic settings based on the insights gained. By bridging the gap between previous literature and our findings, we contribute to the ongoing dialogue on the validity, limitations, and applications of AFC. These insights are valuable for improving the interpretation of facial expressions in real-life settings. Future studies should continue integrating wearable headcams, exploring complementary techniques, and refining AFC algorithms to effectively handle the complexities of naturalistic expressions, in order to further advance our understanding of emotional expressivity in diverse contexts.

### 4.4. Gender differences in parent expressions

Applications of automated facial coding in parenting research have been minimal; we identified only a handful of studies using videos of mothers (Mahoor et al., 2009; Messinger et al., 2009; Haines et al., 2019; Karreman and Riem, 2020; Lyakso et al., 2021) and none using videos of fathers. Without these father-specific studies, nobody has yet linked automated facial coding and parent gender; we believe that our work is the first. Previous studies (Terzis et al., 2010; Lewinski et al., 2014) have shown that automated facial detection using the FaceReader software can vary by gender. Thus, it is not surprising that our models showed gender-related differences in performance, with the fathers’ models exhibiting slightly higher accuracy, specificity, and sensitivity compared to the mothers. To reflect on the disparities in model performance, we must also examine the similarities and differences between the mother and father datasets.

Looking first at similarities, we used an almost identical number of participants of each gender (13 fathers, 14 mothers). Both sets of parents were recruited via the same longitudinal cohort study, and attended assessment clinics within a year of infant birth. The parents also followed identical video recording procedures: they were given headcams, and asked to use them to record multiple interactions with their infant at home. As such, the interactions were consistently naturalistic in structure.

In terms of differences, we believe that many examples are present within the videos themselves. For example, a higher proportion of mothers were wearing glasses in the videos compared to the fathers. This could explain why we observed decreased performance in the mother models (which was modulated to a degree in the combined models). It is well established that glasses cause detection issues in FaceReader (Loijens et al., 2020), so it would not be surprising if the impact of this imbalance was reflected in our model performance. Additionally, our data pre-processing removed a higher percentage of data where a second person was present in the frame for the father dataset compared to the mothers (the father data was reduced by 91%, the mother data reduced by 88%). This could mean that whilst the fathers were recording their videos, it was more likely that another person was present somewhere nearby. It is possible that the presence of another person altered the fathers’ actions and expressions.

We also observed differences in the types of interactions recorded by both fathers (67% feeding, 28% free play and 5% combination) and mothers (66% feeding, 20% free play and 14% stacking). Although the proportion of feeding interactions was consistent, the fathers engaged in more free play, and the mothers included an additional interaction type. Considering the potential impact of different interaction types on parent (and infant) movements, body orientation and positions, future work could explore the use of an interaction term to investigate how interaction type influences the performance of models. The changing orientations during different interactions may pose challenges for FaceReader in accurately detecting facial expressions.

Table 5 showed a summary of the expression intensities (as quantified by FaceReader) in mothers and fathers, revealing that mothers exhibited higher intensities of Happy, Sad, Surprise and Disgust compared to fathers. The mean intensities for Neutral, Angry, Scared and Contempt expressions were similar between mothers and fathers, with generally low intensities observed for these emotions in both groups. This is consistent with previous literature that found that men are less emotionally expressive (Campos et al., 2013), display less intense happy expressions (Oveis et al., 2009) and smile less than women (LaFrance et al., 2003).

Figure 1 highlighted that whilst there was a similar balance of facial expressions across the mother and father datasets, the percentages were not identical. Some expressions occurred more in the father dataset—i.e., Positive and Negative—whilst others occurred more in the mother dataset—i.e., Neutral and Surprise. Whilst these differences may in part explain the variation in model performance between genders, they might also explain some of the values of the gender coefficient in our combined logistic regression models. For example, we saw that: (1) there was a higher percentage of Negative in the father dataset, and (2) the gender coefficient in Table 6 indicated that father’s faces were predictive of Negative expressions. As another example, Neutral occurred more in the mother dataset, and the gender coefficient in Table 6 indicated that mother’s faces were more predictive of Neutral expressions.

Following a comparison of these datasets and results, we are well placed to discuss the future of linking automated facial coding and parent gender. Future studies should continue to investigate applications of automated facial coding to parents (regardless of gender), aiming for consistency in study processes in order to evaluate any gender differences where possible. Maintaining consistency in recruitment procedures, for example, is important to prevent inherent biases in the data (e.g., population biases from recruiting in certain geographic regions). To better assess gender influence, future studies should aim for consistency between videos, by: recruiting an equal number of glasses-wearing mothers and fathers, using the same interaction types for the two genders, and collecting data on other people present during filming. By addressing these factors, researchers can remove their impact on models to obtain more reliable results.

### 4.5. Strengths

The biggest strength of our work is the inclusion of real-life videos. This meant that our interactions were varied, complex and dynamic, capturing a range of ecologically valid, unposed facial expressions. Previous research has also highlighted that interactions recorded at home are more likely to contain less socially desirable behaviours (Lee et al., 2017). Further, by using headcams to record the videos, we captured a closer, more full view of parents’ faces, as opposed to if we had used stationary cameras (which can miss capturing facial expressions; Lee et al., 2017). Another strength is the inclusion of both mother and father videos. As facial expressivity has been studied more in women than men, it was advantageous to include both genders in this work for the purposes of evaluating differences.

Finally, both our participants and double coders were blind to our research aims, meaning that they were not influenced by expectations of the study. For the coders, this helped to improve validity of the coding (facial expressions were analysed based solely on the data in the videos), and enhanced the objectivity of our research process. For the participants, this increased the validity of recordings, as participants would not (knowingly or otherwise) modify their facial expressions to align with the study expectations, leading to more natural behaviours.

### 4.6. Limitations

Our data showed a poor balance of facial expressions, which is not unusual since certain expressions are likely to be less common in parent-infant interactions (e.g., Negative). Although we used a weighting parameter in the models to account for imbalanced classes, providing more data for some expressions may have improved model performance.

It is likely that our data contained biases. Firstly, the length of video material provided by each participant was varied, and these imbalances might have led to biases in model training. Although we mitigated this effect by ensuring that all data from a single participant was contained in either the training or the test dataset. Secondly, the cohort study from which our participants were recruited (ALSPAC) likely contains biases that potentially reduce the representativeness of the sample (Lawlor et al., 2019). For example, there is a high percentage of White-European participants, meaning our findings may not be generalisable to the wider population.

Facial recognition algorithms are prone to racial biases (Buolamwini and Gebru, 2018), especially for dark-skinned females. This bias is due to the lack of diversity in training datasets, which mostly feature light-skinned individuals. The Karolinska Directed Emotional Faces dataset (Lundqvist et al., 1998), used to train FaceReader, comprises models of European descent. Although the software includes an ethnicity classifier, the creators highlight the need for adjustments to better detect expressions on dark-skinned people (Loijens et al., 2020).

The manual and automated coding categories used in this study did not align perfectly, as indicated in Table 1. In order to address this discrepancy, we aggregated the categories into four overall categories that were mutually exclusive. However, this approach may have resulted in a potential loss of valuable information. Specifically, the aggregation of the manual codes ‘Smile’ and ‘Positive’ into the singular category of ‘Positive’ overlooks the meaningful distinction between these expressions (Costantini et al., 2021). Smile involves engagement of both the eyes and the mouth, whilst positive expressions are limited to the mouth and encompass positive interest and excitement. Maintaining the original categories would have allowed for a more nuanced analysis, particularly regarding the differences in intensities. Another example is the differentiation between Mock surprise and Surprise, which are distinct expressions (Costantini et al., 2021). Mock surprise represents a “posed” surprise, whilst Surprise captures genuine reactions. Retaining the original categories would have provided a more comprehensive understanding of these expressions and their relationship to parent responses and emotional communication. Nevertheless, despite the need for aggregation to align with the facial expressions detected by the automated coding software, it remains valuable to employ facial recognition technology to quantify and explore the nuances within these original expressions. This approach can provide insights into the captured variations and contribute to a deeper understanding of parent-infant interactions.

The final limitation of our study is that parents’ expressions may have been affected by the headcams. For instance, parents might have reacted positively to seeing their infant wear the headcam, or might have frowned if the headcam placement was distracting. However, we believe including ecologically valid expressions from real-life interactions mitigated this effect.

### 4.7. Conclusion

In summary, our work investigated the use of an automated facial coding software as an alternative to manual facial coding (which is time intensive, and may be biassed). Our analyses used real-life videos of parents, captured using wearable headcams during interactions with infants in the home. We found that the software classified parent facial expressions around 25% of the time, and the software outputs predicted manually coded expressions with relatively high accuracy, sensitivity, and specificity. Our findings also highlighted which expressions were more or less likely to occur depending on parent gender.

Our work is novel in its inclusion of unstructured, naturalistic observations, captured using headcams; previous studies have used structured recordings, captured using webcams or other stationary cameras. These procedures ensured that our videos were representative of real-life conditions, and that our participants displayed natural, unposed facial expressions. Whilst this led to a reduced automated facial detection rate, we were able to evaluate reasons for this in order to provide robust, evidence-based recommendations for future researchers.

Finally, automated facial coding has only been applied to videos of mothers in a handful of studies (and never to fathers); ours was the first study to use an automated facial coding software to analyse both concurrently. This allowed us to explore the influence of gender, and discuss how future work should modify data collection to promote greater consistency between father and mother videos (allowing for an improved comparison). As we set a benchmark in examining FaceReader gender differences, future studies can build upon our work to further explore this area.

Overall, our research not only advances the field of facial coding but also offers valuable insights into improving the efficiency and accuracy of automated methods.

## Data availability statement

The datasets presented in this article are not readily available because restrictions apply to the availability of these data, which were used under licence for the current study, and so are not publicly available. However, the data that support the findings of this study are available from the Avon Longitudinal Study of Parents and Children (http://www.bristol.ac.uk/alspac/) upon approval from the ALSPAC Executive Committee. Requests to access the datasets should be directed to alspac-data@bristol.ac.uk.

## Ethics statement

The studies involving human participants were reviewed and approved by ALSPAC Ethics and Law Committee. The patients/participants provided their written informed consent to participate in this study. Written informed consent was obtained from the individual(s) for the publication of any identifiable images or data included in this article.

## Author contributions

RP and ICu: data collection. RB and ICo: video coding. RB, ICo, and RP: video processing. RB, IN, RP, and HB: data analysis. RB: manuscript. All authors contributed to the article and approved the submitted version.

## Funding

The UK Medical Research Council and Wellcome (Grant ref: 217065/Z/19/Z) and the University of Bristol provide core support for ALSPAC. This publication is the work of the authors; RB, ICu, ICo, and RP will serve as guarantors for the contents of this paper. A comprehensive list of grants funding is available on the ALSPAC website (http://www.bristol.ac.uk/alspac/external/documents/grant-acknowledgements.pdf). This work is part of a project that has received funding from the European Research Council (ERC) under the European Union’s Horizon 2020 research and innovation programme (Grant agreement No. 758813; MHINT). RB was supported by the Engineering and Physical Sciences Research Council (EPSRC) Digital Health and Care Centre for Doctoral Training (CDT) at the University of Bristol (UKRI Grant No. EP/S023704/1). Dr. Culpin was supported by the Wellcome Trust Research Fellowship in Humanities and Social Science (Grant ref: 212664/Z/18/Z).

## Conflict of interest

The authors declare that the research was conducted in the absence of any commercial or financial relationships that could be construed as a potential conflict of interest.

## Publisher’s note

All claims expressed in this article are solely those of the authors and do not necessarily represent those of their affiliated organizations, or those of the publisher, the editors and the reviewers. Any product that may be evaluated in this article, or claim that may be made by its manufacturer, is not guaranteed or endorsed by the publisher.

## Footnotes

^1^ https://www.bristol.ac.uk/alspac/researchers/our-data/
